# 10-year follow-up of the Columbus knee prostheses system in a prospective multicenter study

**DOI:** 10.1007/s00402-021-04156-9

**Published:** 2021-09-09

**Authors:** Andreas Fuchs, Philip Häussermann, Dirk Hömig, Björn Gunnar Ochs, Tim Klopfer, Christof A. Müller, Peter Helwig, Lukas Konstantinidis

**Affiliations:** 1 Department of Orthopedics and Trauma Surgery, Faculty of Medicine, Medical Center, Albert-Ludwigs-University of Freiburg, Hugstetter Straße 55, 79016 Freiburg, Germany; 2 Clinic for Orthopedics and Trauma Surgery, Sana Kliniken Leipziger Land, Borna, Germany; 3 Clinic for Orthopaedic Surgery, Ortenau Klinikum, Offenburg, Gengenbach, Germany; 4 Vincentius Orthopaedic Clinic, Konstanz, Germany; 5 Clinic for Trauma Surgery, BG-Klinik Tübingen, Tübingen, Germany; 6 Clinic for Trauma, Hand and Orthopaedic Surgery, Städtisches Klinikum Karlsruhe gGmbH, Karlsruhe, Germany; 7 Clinic for Orthopedics and Trauma Surgery, Klinikum Heidenheim, Heidenheim, Germany

**Keywords:** Total knee replacement, Long-term outcome, Prospective multicenter study

## Abstract

**Introduction:**

As endpoint of a prospective multicenter 10-year documentation using the Columbus system, this evaluation carried out results of clinical scores (Knee Society Score and Oxford Knee Score), an evaluation of radiological imaging, survival rates and a collection of complication statistics.

**Materials and methods:**

There was a multicenter prospective recruitment of consecutive patients with the indication for total knee replacement (TKR). Preoperatively and 10 years after implantation, clinical scores, range of motion and radiological imaging was performed. During this period, a detailed documentation of complications was made.

**Results:**

A total of 210 patients were recruited in 5 centers. 140 patients were available for endpoint examination 10 years after surgery. A survival rate of 96.6% (CI 95%) for the implanted Columbus prostheses after 10 years was demonstrated. Cumulative KSS showed an improvement of 75.3 (± 38.1) points and was highly significant (*p* < 0.0001, *t* test). The average functional improvement in the Oxford score was 20.6 (± 9.5) points and was also highly significant (*p* < 0.0001). The ROM improved from 106.5° (± 20.5) preoperatively to 116.0° (± 11.5) (*p* < 0.0001, *t* test). There were no implant-related complications as well as no new complication documented between 5- and 10-year follow-up.

**Conclusions:**

The endpoint analysis after an observation period of 10 years provided good clinical and radiographic results. In particular, an excellent survival rate of 96.6% after 10 years was demonstrated. The data published in this study are the first to be available in a prospective multicenter study on this system, which leads to a high level of clinical significance.

## Introduction

In the past 4 decades, knee arthroplasty has become an increasingly successful surgical treatment for degenerative or arthritic joint diseases, with subjective patient satisfaction having improved significantly [25]. Thanks to the further development of implants used in modern arthroplasty, also younger patients with higher activity levels can be treated satisfactorily. In view of these developments, the performance in terms of clinical and radiological outcome, as well as the survival of the implanted models are in focus of scientific work [10, 11, 17, 19]. The Columbus knee endoprosthesis system (Aesculap—Tuttlingen) was released in 2003, the implantation can be performed either manually using the conventional instruments or using the Ortho-Pilot navigation system. Furthermore, an implantation according to the “tibia-first” as well as the “femur-first” method is possible, whereby simple instruments enable a high reproducibility of the results [10, 15, 30, 31].

The current literature shows a wide range of evidence regarding the clinical and radiological outcome after implantation of a Columbus knee endoprosthesis, but most of them present short-term follow-up. Mid- and long-term performance data are rare, so that there is currently no sufficient and homogeneous evidence [1, 3, 9–11, 22, 26, 31].

The aim of this study was to generate data of an observation period of 10 years after implantation of a Columbus total knee joint endoprosthesis. Survivorship as well as clinical and radiological outcome parameters were evaluated and analyzed in particular with regard to the data from various endoprosthesis registers. This survey represents the end point of a prospectively multicenter study. Intermediate surveys were carried out after 1 year and after 5 years.

## Materials and methods

### Study protocol

In this prospective multicenter (Level III) study, patients with indication for TKR were consecutively included in the years 2006–2009. The decision for surgery was made with no regard to the current study. The study design was prospective from the start with the intention of tracking the included patients over a period of 10 years. Patients were recruited in five centers with different care assignments. To ensure the longest possible follow-up period, a maximum patient age of 72 years at the time of the operation was specified for the recruitment of patients for this prospective study. Furthermore, for the same reason, only patients classified as ASA I or ASA II (according American Society of Anesthesiologists) were recruited. Patients with a mechanical leg axis between 20° varus and 15° valgus and a maximum flexion contracture of 20° were included. A deviation of leg axis beyond these values led to an exclusion of the patient for the study. Further exclusion criteria were: previous fractures involving the knee joint or previous osteotomies, bone metabolism disorders (with the exception of osteoporosis), alcohol addiction, Parkinson’s disease and systemic malignancies. After reviewing the inclusion and exclusion criteria, as well as the patient’s consent (approval by the Freiburg Ethics Committee International—feki code 06/1609), the preoperative assessment of clinical scores below and the evaluation of radiological parameters mentioned below were carried out. The study was sponsored by Aesculap AG.

A total of 210 patients in 5 centers were included to the study. The main indication for total knee replacement in the patient population included was osteoarthritis (OA) of the knee (200 patients, 95.2%). Secondary, OA of post-traumatic origin or other cases of secondary OA were reported. In 151 patients, surgery was performed using navigation software (Orthopilot—Aesculap), 59 patients were treated without navigation support. In three cases, patella back surface replacement was performed as part of the primary TKR surgery. 59% of the patients included in the study were female, 41% of the patients were male. In 52% of the cases, the knee joint endoprosthesis was implanted on the right side, in 48% on the left side. The demographic data of all patients included in the study are listed in Table 1.

**Table 1:** Demographic data of study population

	Mean	StdDev	MIN	MAX
Age (y)	63.10	6.63	36	72
Weight (kg)	86.37	15.71	50	133
Height (cm)	168.44	9.19	145	193
BMI (kg/m^2^)	30.48	5.36	20.55	48.85

### Surgical technique

The implantation of the investigated TKR system (Columbus CR, Deep Dish fixed inlay, Aesculap—Tuttlingen) was performed both navigated (three centers) and manually (two centers). The decision between navigated or manual implantation was solely dependent on the clinical routine in the respective clinic. Deep dish inlays were used for implantation in all study participants, as it represents the standard inlay for Columbus users and was routinely implanted in the recruiting clinics regardless to the study. Both, tibial and femoral components were implanted with cement (Palacos R + G, Heraeus Kulzer—vacuum cementing system). The cementation (vacuum technology) was only carried out as surface cementation, i.e. the cement was only placed on the surface of the implant and not in the medullary canal. All implantations were performed according to the tibia-first technique.

### Clinical scores

Clinical scores were obtained for all patients included to the study preoperatively and 10 years after surgery, the examination included Knee Society Score (KSS) and the Oxford Knee Score [7, 14]. The KSS includes both objective and subjective parameters and is divided into “Knee Score” with recording of the parameters pain, range of motion (ROM), stability, contracture and radiological alignment, as well as “Function Score” with documentation of the activity and walking aids. The Oxford Knee Score consists of 12 questions and only takes anamnestic factors into account. In the KSS Score, a higher score represents a better result, whereas the Oxford Score shows a better outcome with a lower score.

### Radiological imaging

After inclusion of the respective patient, the following radiological parameters were evaluated preoperatively on the basis of study-independent X-rays of the knee joint on two planes (anteroposterior and lateral): the anatomical leg axis, the mechanical axis and the lateral distal femoral angle (LDFA) were evaluated on the basis of whole-leg X-ray imaging. Values of the mechanical leg axis  < 180° corresponded to a valgus deformity,  > 180° to a varus deformity. The LDFA was measured laterally as the angle between the mechanical femoral axis and the joint line.

As part of the radiological follow-up, knee joint X-ray imaging (anteroposterior and mediolateral) were used to evaluate “radiolucent lines” in the corresponding zones according to Knee Society Total Knee Arthroplasty Roentgenographic Evaluation and Scoring System [8]. The anatomical and mechanical leg axis as well as the LDFA were determined on the follow-up whole-leg X-rays in accordance with the preoperative protocol.

### Complication statistics

The occurrence of any complication was documented in detail in the corresponding form. In the event of a revision operation, which led to an exchange of a prosthetic component, the patient was excluded from the study.

### Statistical analysis

The data of clinical scores, radiological imaging and survival rates were assembled into a database and analyzed using SAS^®^ software (version 9.4, SAS Institute Inc., Cary, NC, USA). Data were reported as means and standard deviation (SD). Survival rates were reported using Kaplan–Meier and competing risk analysis. Comparisons between groups were done by paired *t* test. Differences between groups were considered to be significant if *p* < 0.05.

This 10-year follow-up represents the endpoint of clinical evaluations after 1, 5 and 10 years, the data of 1-year and 5-year follow-up were also implemented in the statistical analysis.

## Results

### Survival and complications

**Fig. 1:**
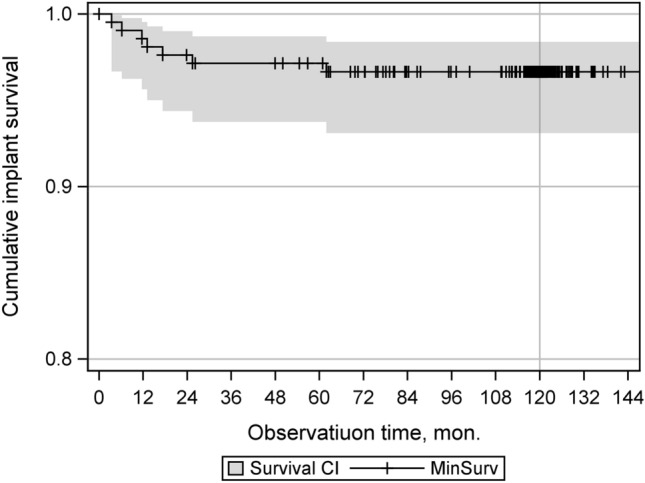
Kaplan–Meier plot of cumulative implant survival over follow-up period (months)

Of the 210 patients recruited at the time of primary implantation, 140 (67%) could be included in the evaluation of the 10-year follow-up. This shows a dropout of a total of 70 patients, with 18 patients dying during the follow-up period and 14 patients withdrawing their consent to participate in the study without giving any further reasons (Fig. 1).

**Fig. 2:**
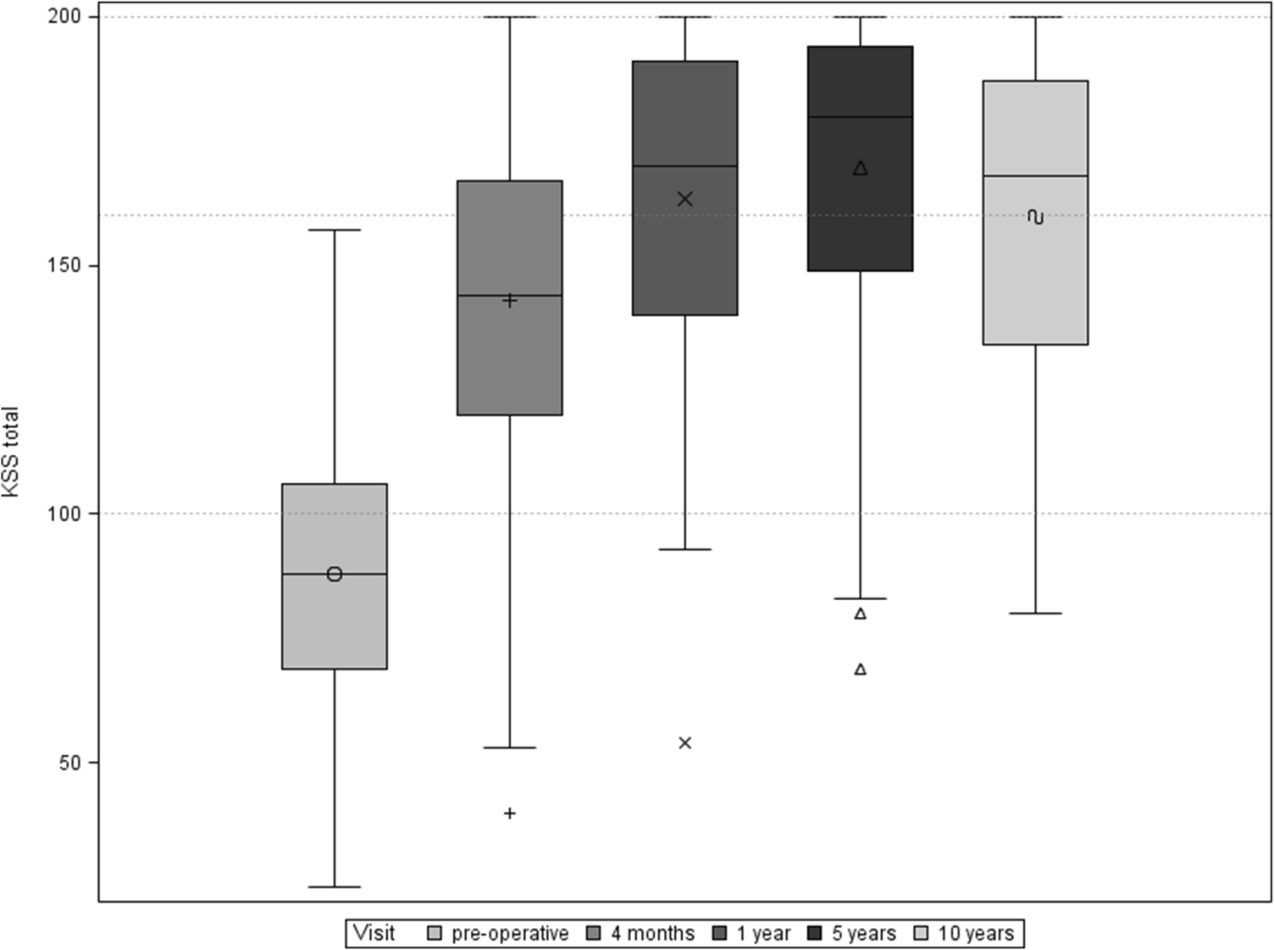
Flowchart of study population with reason for study termination

During the entire follow-up period of 10 years, a total of seven revision operations were carried out. There was no prosthesis-removal documented due to aseptic loosening or ligament instability. This corresponds to a 10-year survival rate of the Columbus knee joint endoprosthesis of 96.6% (93.1, 98.4) with the end point of prosthesis- or inlay change  (Fig. 2). The competing risk analysis shows a failure rate in the follow-up period of 3.35% (1.6, 6.9). The worst-case survival, taking into account all dropout cases, including deaths, is 65.6% (61.4, 69.8).

The complication statistics show a total of 34 cases. This includes both revision operations with replacement of prosthesis components (*n* = 7) and other reoperations (*n* = 6). There was also a discrimination between perioperative complications (*n* = 3), general complications (*n* = 4), and postoperative complications that occurred during inpatient stay (*n* = 9) and post-inpatient (*n* = 5). Perioperative complications include two tibial fissures and an immediate postoperative hypesthesia in the area of the scar. General complications were a urinary tract infection, a clinically manifest deep vein thrombosis, an apoplexy and a cardiac complication with cardiac arrhythmias. Postoperatively, there were two cases of wound healing disorders, four hematomas, one deep infection, one hypesthesia in the surgery area and one scar neuroma of the ramus infrapatellaris of the saphenous nerve. Post-inpatient inadequate ROM manifested in three cases, one case of pain persistence and one complication in connection with a knee orthesis. There were no implant-related complications such as inlay dislocation, material failure or aseptic loosening. Additionally, no new complications were observed between the 5-year follow-up and the 10-year follow-up examination.

### Clinical scores

**Fig. 3:**
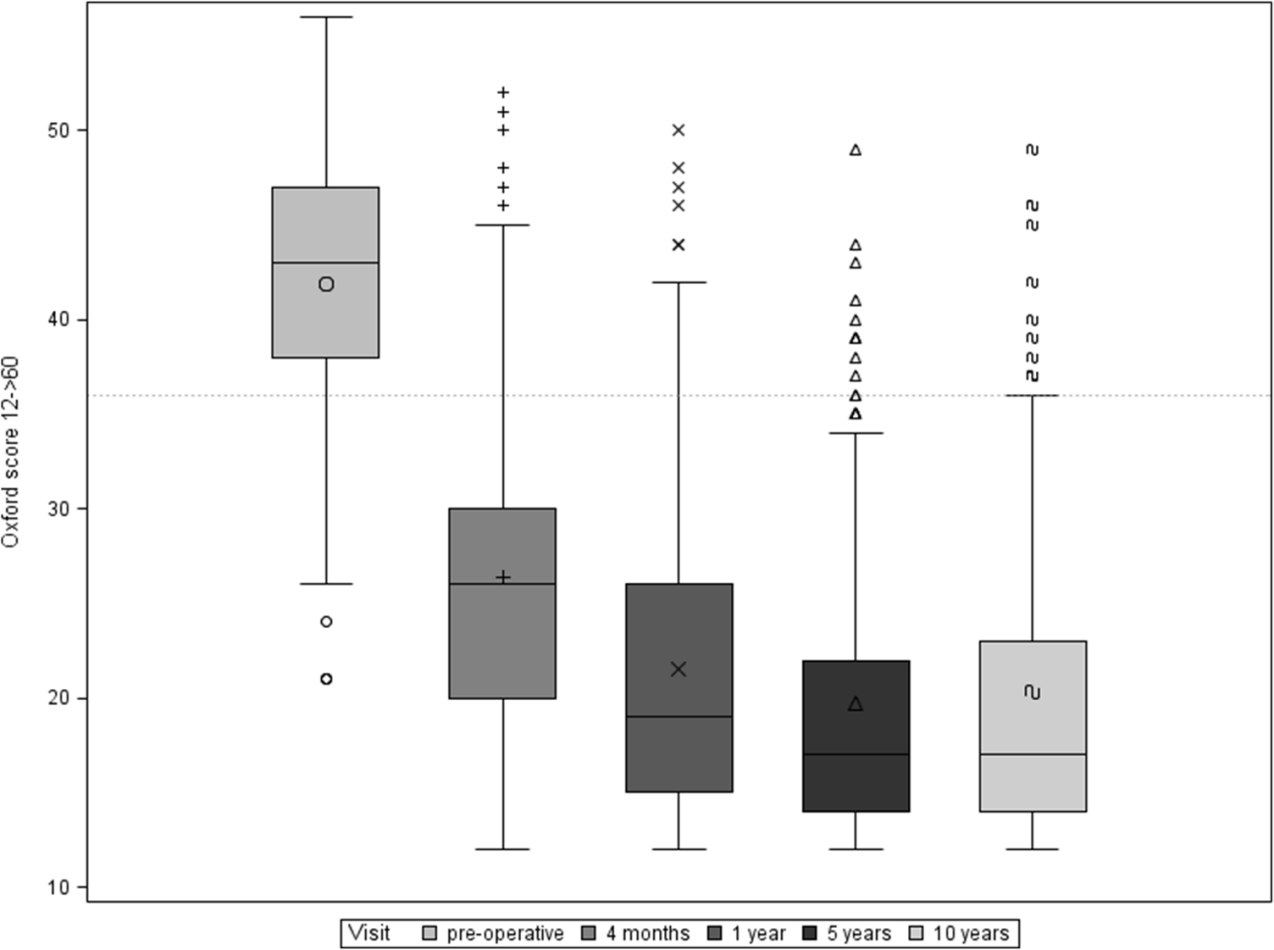
Boxplot of cumulative KSS over follow-up period

Knee Society Score The cumulative KSS increased from 87.5 (SD ± 26.6) preoperatively to 160 (SD ± 30.3) 10 years postoperatively (Fig. 3). This corresponds to an improvement in the cumulative KSS of 75.3 (± 38.1) points in the mentioned follow-up period, which is statistically highly significant (*p* < 0.0001, *t* test). The increase in the individual sub-scores of the KSS (“knee” and “function”) was correspondingly significant. The KSS knee increased from 38.1 (SD ± 17) preoperatively to 86.0 (± 12.1) 10 years postoperatively (*p* < 0.0001, *t* test). The KSS function improved from 49.8 (SD ± 17.3) preoperatively to 74.3 (± 24.9) 10 years postoperatively (*p* < 0.0001, *t* test).

**Fig. 4:**
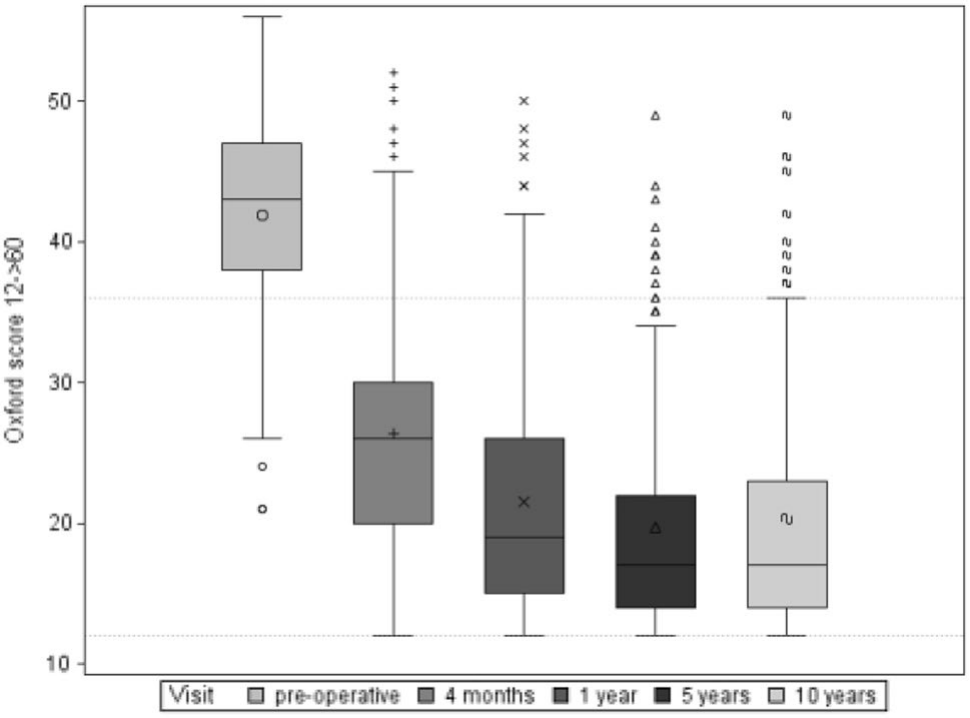
Boxplot of cumulative Oxford Score over follow-up period

Oxford Knee Score: The Oxford Score evaluated in the study population improved from 41.8 (SD ± 6.7) preoperatively to 20.3 (SD ± 8.9) 10 years postoperatively (Fig. 4). The mean subjective improvement in function between the preoperative time and the 10-year follow-up was 20.6 (SD ± 9.5) points, which also represents a statistically highly significant improvement (*p* < 0.0001).

Range of Motion [27]: A statistically highly significant improvement in the range of motion (ROM) from 106.5° (± 20.5) preoperatively to 116.0° (± 11.5) 10 years postoperatively (*p* < 0.0001, *t* test) was detected.

### Radiological evaluation

#### Mechanical leg axis

**Fig. 5:**
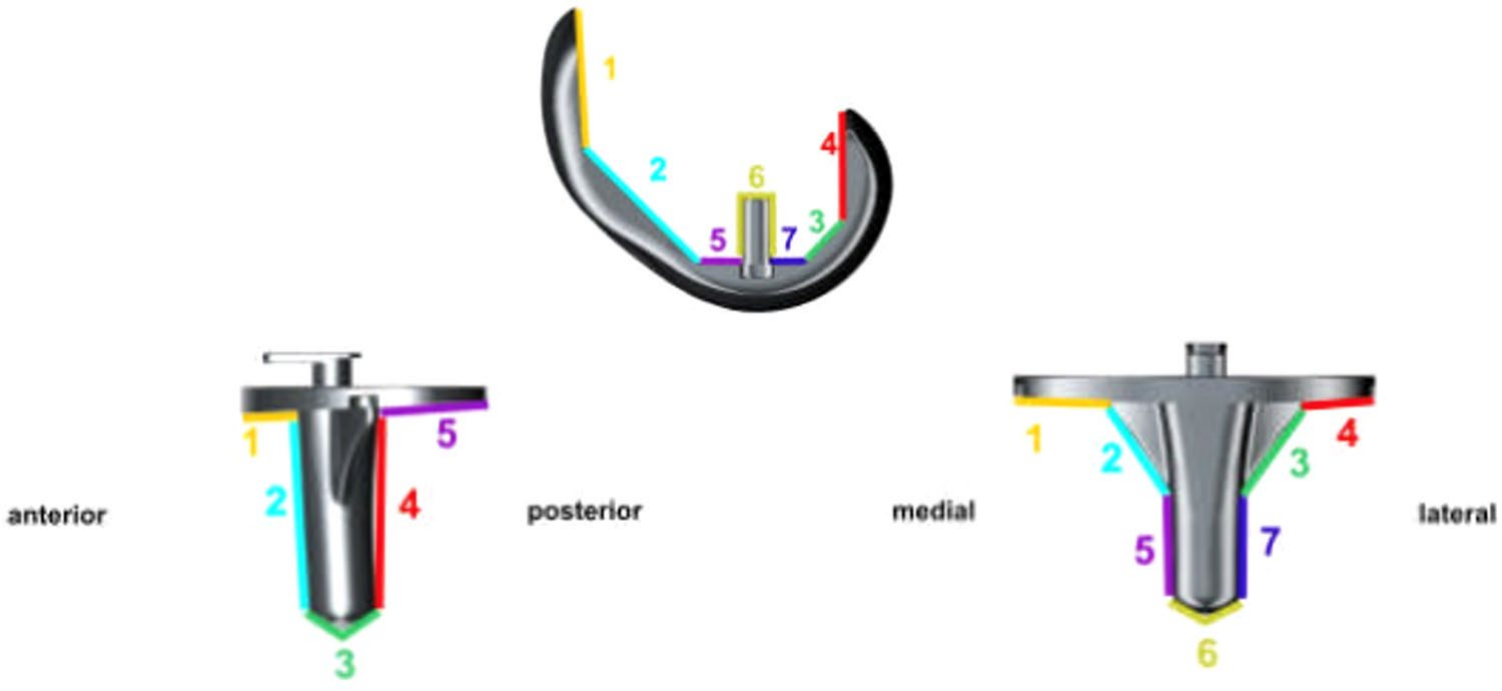
Schematic representation of radiolucent lines

The mechanical leg axis in the study population was 175.1° preoperatively (SD ± 5.7°), which corresponds to an average genu varum of 4.9°. 10 years after total knee replacement surgery, an average mechanical leg axis of 178.7° (SD ± 2.8°) was shown, corresponding to a regress of varus of 3.6° compared to the preoperative situation. 55 patients (26.8%) showed a mechanical leg axis deviation of ± 3° preoperatively. 10 years after surgery, 83 patients (74.8%) showed a mechanical leg axis with a maximum deviation of 3° varus or valgus. In the endpoint evaluation, 20 patients (18%) showed a mechanical leg axis within an axis deviation of 3–5° (varus or valgus) and 8 patients (7.2%) were identified with an axis deviation of more than 5°. The anatomical leg axis was evaluated as part of the KSS and is, therefore, reflected in the analysis of the clinical scores (Fig. 5).

#### Lateral distal femoral angle (LDFA)

In addition to the mechanical leg axis, the LDFA was also evaluated. As part of the surgical technique performed, a postoperative angle of 90° between the femoral joint line and the mechanical axis of the femur was aimed for. The average LDFA preoperatively was 90.6° (SD ± 2.9), 10 years postoperatively the mean value was 89.4° (SD ± 2.3°).

#### Radiolucent lines

In the postoperative analysis of radiolucent lines and according to the Knee Society Total Knee Arthroplasty Roentgenographic Evaluation and Scoring System, a measuring of the width of radiolucent lines in defined zones was performed [8]. Three categories were defined: 1st radiolucent lines  < 1 mm, 2nd radiolucent lines 1–2 mm, third radiolucent lines  > 2 mm.

In a total of three tibial prosthesis components, a width of the radiolucent lines of  > 2 mm was found in the 10-year follow-up. In the ap projection, these were found in zones 4 and 7, in the side view in zone 1 according to the Knee Society Total Knee Arthroplasty Roentgenographic Evaluation and Scoring System [8].

In the analysis of the femoral prosthesis components, we identified six components with a width  > 2 mm, each of which was found ventrally on the femoral shield. In five cases, the radiolucent lines appeared in zone 1, in one case in zone 2.

Despite of the evidence of some radiolucent lines corresponding to category 3 in a few defined zones, there was no clinically manifest or radiologically visible aseptic loosening of the femoral or tibial joint component.

## Discussion

The aim of this study was to provide data about survival statistics as well as clinical and radiological outcome after the implantation of a Columbus total knee endoprosthesis over a period of 10 years. This survey represents the end point of a prospective multicenter study. Intermediate surveys were carried out after 1 year and after 5 years. A comparison of results between the intermediate surveys, especially the 5-year follow-up, and the endpoint evaluation should also be demonstrated.

The data collected in this study show a 10-year survival rate of 96.6% with the end point of prosthesis- or inlay change. The intermediate survey after 5 years showed a survival rate of 97.1% [9]. With regard to the above-mentioned highly significant improvements in all measured clinical scores and an excellent survival rate of 96.6% 10 years after implantation, as well as the constantly satisfactory results compared to the intermediate surveys within this study, a very good long-term performance of the Columbus system can be postulated.

To compare the results of this study with those of prosthesis models with similar geometry and kinematics, clinical studies of the current literature on the well-established PFC Sigma prosthesis (DePuy Synthes) were investigated.

Dalury et al. published the results of 1970 PFC Sigma total knee endoprostheses implanted in a multicenter retrospective study, which were implanted between June 1996 and December 1997. 1316 prostheses had a follow-up period of 5 years or more. The authors postulated a 10-year survival rate of the implanted prosthesis of 95.6% (CI 95%). From the data obtained, the authors attested excellent results of the PFC Sigma prosthesis in the medium- and long-term follow-up [6].

In a comparative study of the PFC Sigma CR mobile bearing prosthesis with long-term follow-up, Kim et al. published a survival rate of 99.5% (CI 95%) after an average follow-up period of 12.6 years [16].

As part of its annual report, the UK National Joint Registry published 2019 an average 5-year revision rate of the PFC Sigma Bicondylar Knee system of 1.96%. After 10 years, there was an average revision rate of 2.74% and after a follow-up of 13 years of 3.20%.

With regard to the Columbus system, revision rates of 2.33% after 5 years, 3.28% after 10 years and 3.81% after 13 years were published [20]. In summary, the comparison of long-term follow-up performance between the two established systems (PFC Sigma and Columbus) show comparable survival rates of the implanted prostheses, both on the basis of the current literature and in relation to the population examined in this study.

In contrast to the majority of data available in international literature, the Australian prosthesis register published 2016 significantly different data on the PFC Sigma—compared to the Columbus system. The register data showed revision rates for the cemented PFC Sigma CR system of 2.4% after 5 years, 2.9% after 7 years and 3.4% after 10 years. Data regarding the Columbus system could only be found in the register for the non-cemented option. This showed an average revision rate of 9.8% after 5 years and 11.4% 7 years after primary implantation [1]. In a prior publication of 5-year data of this study population, these findings were already discussed controversially [9]. While detailed data for the PFC Sigma prosthesis were published when using the cemented version, revision statistics for the Columbus prosthesis were only based on non-cemented prosthesis models. The authors concluded that a bias of the published statistics with regard to possible unsuitable use (e.g. cementing in non-cemented prosthesis models) or a learning curve for the surgeons cannot be ruled out [9].

The Australian Orthopaedic Association National Joint Replacement Registry (AOANJRR) Annual Report 2019 also provides data regarding the Columbus system for the cemented implantation option. Here, revision rates of 0.6% after 1 year, 2.4% after 5 years and 3.0% after 10 years were published [2]. These findings finally confirm both, the consistently satisfactory data in international literature and the results obtained in the context of this study.

The clinical data collected in this study show an improvement in ROM from 106.3° (SD ± 20.2°) preoperatively to 116.0° (± 11.5) 10 years postoperatively. Furthermore, there was a highly significant improvement in both the cumulative KSS and the Oxford Score 10 years after surgery. Compared to the mid-term results after 5 years, which were generated within this prospective study, we see comparable and constant results. After 5 years, the cumulative KSS increased from 87.5 (SD ± 26.6) preoperatively to 170 (SD ± 29.1), and the Oxford Score improved from 41.8 (± 6.7) preoperatively to 19,4 (SD ± 7.6) [9]. These findings support the good long-time performance of the Columbus system, slight differences between 5-year und 10-year KSS and Oxford Score can be explained with increasing age of the study population. We do not conclude a decreasing performance of the implanted Columbus knee prostheses.

A comparison with existing data on the PFC Sigma system is also useful when examining clinical parameters due to the implant design. In a comparative study of the PFC Sigma CR mobile bearing prosthesis with long-term follow-up, Kim et al. showed improvements of KSS from 28.7 points preoperatively to 95 points at the time of the last follow-up examination. According to the publication, the KSS function rose from 44.8 to 80 points in the follow-up examination period. In the clinical study, the ROM also showed an improvement from 123° pre to 128° postoperatively in the long-term follow-up [16].

Luring et al. published a comparison of the mobile and fixed inlay of the PFC Sigma prosthesis with an average KSS of 176 points (SD ± 16) 2 years after implantation with a mean ROM of 112° (± 14°) for the patient collective with fixed Inlay. There were no significant differences between the subgroups [18].

Similar results can be found in a comparative study between fixed and mobile inlay by Hanusch et al. After the first postoperative year, the Oxford Score was 21.4 points (SD ± 7), the KSS knee 84.5 points (SD ± 16.2), the KSS function 76.7 points (SD ± 18) and the ROM at 100° (SD ± 10°) [12].

In a clinical study with an average follow-up of 86 months after implantation of a PFC Sigma—rotating platform CR endoprosthesis, Bhatt et al. showed an improvement of the KSS from preoperatively 53 points to 80 points for the postoperative follow-up. The Oxford Score improved from 43 preoperatively to 21 postoperatively. The authors also published an improvement in ROM in the follow-up period from preoperatively 91° to 113° after an average of 86 months [4].

Current literature also provides some clinical studies with short or medium follow-up using the Columbus system [10, 11, 15, 26, 30]. Stulberg et al. published an average KSS knee score of 88, KSS function score of 76 and a ROM of 120° 2–4 years after navigation-supported implantation of cemented Columbus implants [30]. Schüttrumpf et al. showed 5 year results with a cumulative KSS of 180 (SD ± 16) points under using the cement-free press fit Columbus system and navigation software. The ROM was documented with 116° (SD ± 10°) 5 years after TKR [26].

However, the survey of navigated vs. not navigated TKR was not the primary goal of this study, it should be mentioned that international literature shows no significant difference in functional outcomes and implant survivability between navigated and non-navigated TKA [21]. Furthermore, the cohort analyzed in this study would be too small for a comparison between the groups. For these reasons, no statistical comparative analysis between navigated vs. not navigated TKR was carried out within this study, although both implantation methods were performed in the participating clinics.

One additional interesting aspect of this study was the evaluation of radiolucent lines in standard radiographic projections. In a total of three tibial prosthesis components, a width of the radiolucent lines of  > 2 mm was found in this 10-year follow-up. In the analysis of the femoral prosthesis components, we identified 6 components with a width  > 2 mm, each of which was found ventrally on the femoral shield. In five cases, the radiolucent lines appeared in zone 1, in one case in zone 2 according to the Knee Society Total Knee Arthroplasty Roentgenographic Evaluation and Scoring System [8]. Different aspects in the discussion of radiolucent lines have to be considered. First of all, cementation technique remains a matter of discussion in primary TKA [5, 13, 24, 29, 32]. Here, especially differences between surface and fully cemented components are discussed in literature. Skwara et al. postulated a higher number of failures with fully cemented tibial component compared to a surface cemented component [28] whereas Rossi et al. found comparable results between surface and fully cementation [23]. We identified a conspicuous accumulation of radiolucent lines in zone 1 of the femoral component in our study. In accordance to international literature, we mainly propose that during introduction of the femoral component the cement on the anterior component sheared off which leads to an increase of radiolucent lines in the radiographic follow-up evaluation [29]. Furthermore, these zones underlie a lower axial load which leads to bone resorption and radiolucent lines in the radiographic evaluation. Additionally, we need to take into consideration that only standard radiographs were observed in this study. We did not include any form of functional assessment and, therefore, cannot guarantee the comparability of the investigated radiographs.

Despite of the evidence of some radiolucent lines corresponding to category 3 in a few defined zones, we conclude that there was no clinically manifest or native radiologically visible aseptic loosening of the femoral or tibial joint component in the present study population.

## Limitations

There are some limitations to this study. Initially, only patients with comorbidity up to ASA II were integrated into the study, which leads to a younger and healthier patient population in this study. Such a patient selection could have a negative impact on the result due to the shorter prosthesis survival rates in younger active patients. In addition, very strict complication statistics were kept in which component changes also had an impact on the survival statistics. Additionally, a relatively high dropout rate due to deaths, lost to follow-up and withdrawal of consent must be mentioned. Furthermore, due to the relatively small number of cases, there was no statistical evaluation regarding the comparison of navigated and non-navigated implantation. Finally, no form of functional assessment in radiographic imaging was performed, therefore, the comparability of the investigated radiographs cannot be guaranteed.

## Conclusion

The final evaluation after 10 years as endpoint of the established multicenter, long-term study of the Columbus system showed very satisfactory survival rates as well as clinical and radiological results. Material-related complications or an above average loosening of the implants did not occur. Based on these results and in view of the data available in current international literature, the Columbus system can, therefore, be regarded as a very successful prosthesis system.
