# Correction to: 10-year follow-up of the Columbus knee prostheses system in a prospective multicenter study

**DOI:** 10.1007/s00402-021-04191-6

**Published:** 2021-10-05

**Authors:** Andreas Fuchs, Philip Häussermann, Dirk Hömig, Björn Gunnar Ochs, Tim Klopfer, Christof A. Müller, Peter Helwig, Lukas Konstantinidis

**Affiliations:** 1 Department of Orthopedics and Trauma Surgery, Faculty of Medicine, Medical Center, Albert-Ludwigs-University of Freiburg, Hugstetter Straße 55, 79016 Freiburg, Germany; 2 Clinic for Orthopedics and Trauma Surgery, Sana Kliniken Leipziger Land, Borna, Germany; 3 Clinic for Orthopaedic Surgery, Ortenau Klinikum, Offenburg, Gengenbach, Germany; 4 Vincentius Orthopaedic Clinic, Konstanz, Germany; 5 Clinic for Trauma Surgery, BG-Klinik Tübingen, Tübingen, Germany; 6 Clinic for Trauma, Hand and Orthopaedic Surgery, Städtisches Klinikum Karlsruhe gGmbH, Karlsruhe, Germany; 7 Clinic for Orthopedics and Trauma Surgery, Klinikum Heidenheim, Heidenheim, Germany

## Correction to: Archives of Orthopaedic and Trauma Surgery 10.1007/s00402-021-04156-9

The original version of this article unfortunately contained a mistake. Figure 2 was incorrect.

The corrected Fig. 2 is given in the following page.

**Fig. 2:**
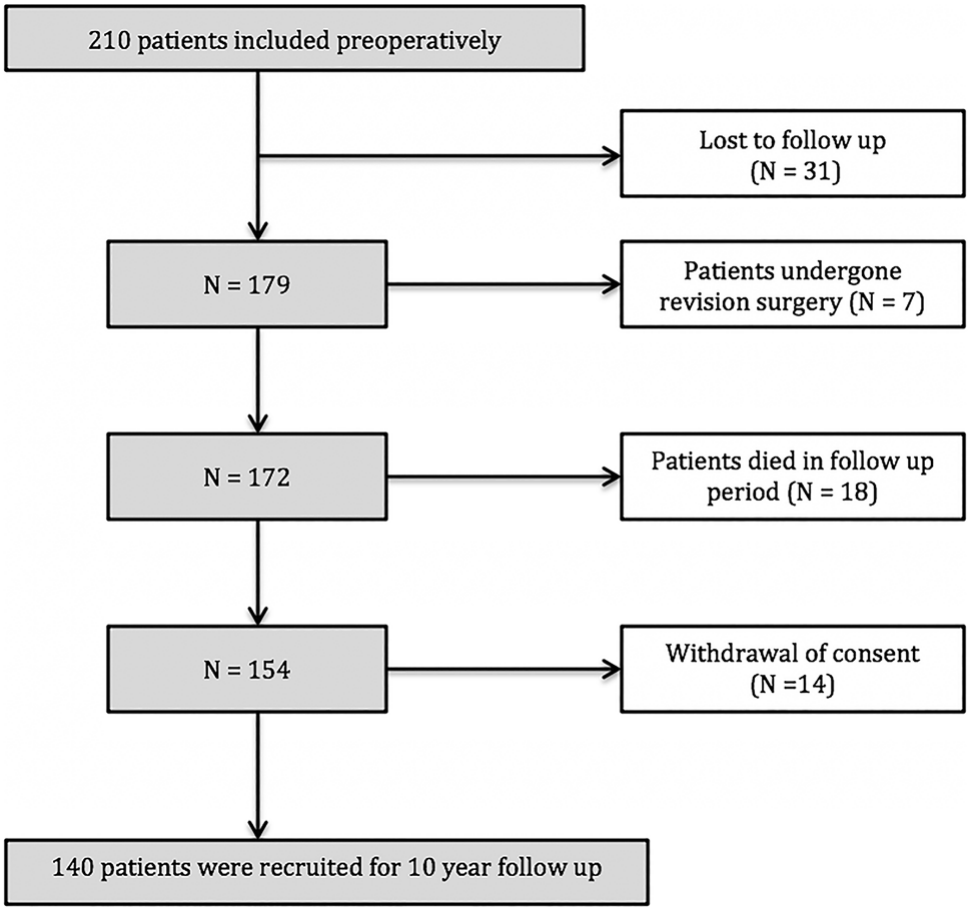
Flowchart of study population with reason for study termination

